# Enrichment of Breadsticks with Flavoured Oils: Chemical Composition, Antioxidant Activity and Technological and Sensory Properties

**DOI:** 10.3390/antiox13121438

**Published:** 2024-11-22

**Authors:** Vincenzo Sicari, Antonio Mincione, Irene Maria Grazia Custureri, Roberta Pino, Monica Rosa Loizzo

**Affiliations:** 1 Department AGRARIA, “Mediterranea” University of Reggio Calabria, Località Feo di Vito, 89124 Reggio Calabria, RC, Italy; vincenzo.sicari@unirc.it (V.S.); irene.custureri@unirc.it (I.M.G.C.); 2 Department of Pharmacy, Health and Nutritional Sciences, University of Calabria, 87036 Rende, CS, Italy; roberta.pino@unical.it (R.P.); monica_rosa.loizzo@unical.it (M.R.L.)

**Keywords:** breadsticks, flavoured oils, bioactive compounds, sensory analysis, texture analysis

## Abstract

The present work compares the physical–chemical, organoleptic and antioxidant characteristics of breadsticks (Bs) prepared in the traditional way (BCs) with extra virgin olive oil (EVOO), and with mace (BMs), ginger (BGs) and turmeric (BTs) flavoured olive oil (FOO). Breadsticks’ water activity (a_w_), pH, moisture content (U.R.), total phenol (TPC) and total flavonoid (TFC) content, colorimetric analysis and texture and sensory analysis were used to evaluate the impact of the new recipes on consumer acceptance. The radical scavenging activity was also assessed by using 1,1-diphenyl-2-picryl hydrazine (DPPH) and 2,2′-azinobis-3-ethylbenzothiazoline-6-sulfonate (ABTS). The use of FOO influenced breadsticks’ colour with reference to the BG and BT enriched breadsticks, and some variability in free acidity values emerged from the comparison between EVOO and FOO. As expected, peroxide values increased in all enriched breadsticks. Moreover, all flavoured breadsticks were more resistant to lipid oxidation than BCs with an IP value of 92.44, 91.26 and 60.07 h, respectively, for BMs, BGs and BTs. The cooking process of the breadsticks at 180 °C for 25 min did not significantly impact the content of bioactive compounds. BMs showed the highest TPC and TFC with values of 996.32 and 534.41 mg/kg, respectively. Moreover, BMs showed the highest DPPH radical scavenging potential with a value of 393.91 µM TEAC/100 g extract, whereas BGs showed the highest ABTS radical scavenging activity (160.13 µM TEAC/100 g extract). Sensory quantitative descriptive analysis showed the most interesting parameters to be the intensity of toasting for BGs and the intensity of spiciness in BMs. Furthermore, BGs and BTs were found to have a slightly more pungent odour. From the texture assessment, the BC was the crumbliest breadstick, while greater crunchiness was found in the BG and BM samples.

## 1. Introduction

Today, food consumers show great attention towards food ingredients. Moreover, the food market shows an increasing demand for foods with a high nutritional profile and for functional foods able to prevent or reduce the risk of the onset of pathologies [1,2].

Breadsticks represent a typical Italian product, shaped as crusty sticks of bread with a brittle and airy texture and a long shelf-life; this product has the potential to capture the preference of modern consumers [3]. Breadsticks are a very common baked snack characterized by a formula in which sugar, wheat flour and virgin olive oil are the main ingredients. Some processes in the preparation of breadsticks, such as refining and bran and germ removal, lead, however, to a reduction in the nutritional value, through losses of proteins, vitamins, minerals and bioactive phytochemicals [4,5].

Extra virgin olive oil (EVOO) represents a key component of the Mediterranean diet.

The habitual consumption of extra virgin olive oil (EVOO) is associated with the prevention of the onset of a series of chronic degenerative pathologies including metabolic syndrome, type 2 diabetes, muscle pathologies, degenerative pathologies of the central nervous system and cancer (e.g., breast and colorectal) [6].

The health potential of EVOO is attributable to the presence of high levels of fatty acids (98–99% of the total weight of oil), in particular monounsaturated acids (17.96%) such as oleic acid and polyunsaturated acids (10.52%) such as linolenic acids. Other bioactive constituents are phenols such as nonpolar oleuropein and ligustroside aglycones and their derivatives, phytosterols and tocopherols [7,8]. Moreover, a single tablespoon of EVOO provides approximately 13% of your daily recommended intake of vitamin E and approximately 9% of your daily recommended intake of vitamin K [9].

From a nutritional point of view, the energy value per 100 g is 884 Kcal, which means there is 125 Kcal in a tablespoon. This suggests that EVOO may be unique among dietary fats in its ability to reduce the risk of multiple chronic diseases and, therefore, its place in dietary guidelines should be emphasized.

Spices and herbs have been in use for centuries both for culinary and medicinal purposes. Spices not only enhance the flavour, aroma, and colour of food, but they can also protect us from acute and chronic diseases. There is now ample scientific evidence that both spices and herbs possess antioxidant, anti-inflammatory, anti-tumour and glucose- and cholesterol-lowering properties. Researchers over the past decade have linked such health properties of spices to their bioactive constituents, including polyphenols, sulphur-containing compounds, alkaloids, di-terpenes, and vitamins [10].

The ginger (*Zingiber officinale*) plant produces an irregular bumpy rhizome, largely used as a spice and for its healthy properties. This spice possesses antioxidant, anti-inflammatory, antimicrobial and anti-tumour activities. Following the advancement in consumer awareness and the industrial demand for organic antioxidants and functional ingredients, the application of ginger and its derivatives has been broadly investigated in a wide range of food products, including bakery, beverage, meat and dairy products [11].

*Myristica fragrans* is a traditional spice largely used in food preparation worldwide. It is composed of two distinctive parts, i.e., nutmeg (seed) and mace (outer covering). Both spices contain significant amounts of bioactive compounds that can reduce oxidative stress, lipid peroxidation and hyperglycaemic conditions [12].

Turmeric is the rhizomatous part of *Curcuma longa*. This species has received much interest for its sensory and health properties. The health properties of this spice range from counteracting the oxidative process to anti-inflammatory activity, thus improving metabolic syndrome and hyperlipidaemia management [13].

Several studies have been conducted to identify matrices especially from the agri-food industry to be added to bakery products with the aim to improve their nutritional and biological value [14,15,16,17,18,19]. These include the addition of ginger, turmeric or nutmeg powder to bread in order to improve the sensory properties and biological activities [20,21].

Several studies have also demonstrated that breadsticks are a suitable target matrix for agri-food by-product fortification. Previous studies evaluated the feasibility of fortifying both traditional and gluten-free breadsticks with olive oil industry by-products, either in their original form or as polyphenol-rich extracts [22,23,24].

Among different EVOO by-products, olive cake powder was found to be the best choice for breadstick fortification in terms of the nutritional and bioactive profiles, as well as an enhanced sensory profile. Besides the effects on consumer preferences, the addition of olive leaves and olive mill wastewater was found to increase the shelf-life and did affect the texture properties of the final products. Breadstick fortification was also achieved with winemaking by-products, such as brewers’ spent grains and grape pomace [25,26], or with artichoke by-products [27].

Furthermore, from a consumer preference analysis associated with breadstick products it emerged that 47% of consumers interviewed would be willing to purchase a pack of breadsticks enriched with phenolic extracts at a higher price than conventional breadsticks, thus recognizing the value of the functional ingredients included in the food formulation [28].

In our continuous research on the development of flavoured olive oils and their new possible applications, in this research we have replaced extra virgin olive oil with flavoured oils in the formulation of breadsticks. Breadsticks are traditional Italian baked products. Based on the literature data and field investigations, we have not found breadsticks obtained by adding flavoured oils on the market. In fact, to date only studies have been carried out that report the use of by-products from olive oil processing [22,29]. We believe that the use of these oils represents a good opportunity for food industries to bring new whole-grain products to market, not only with a healthy potential but also with a longer shelf-life.

For this purpose, we have used olive oil flavoured with ginger, turmeric and mace in the breadstick’s formulation. The resulting products will benefit from the rich content of antioxidant compounds in these spices, not only from the sensorial and shelf-life point of view of the product, but also for its nutraceutical potential, with a positive impact on the health of consumers.

## 2. Materials and Methods

### 2.1. Chemicals and Reagents

Folin–Ciocalteu reagent, gallic acid monohydrate, catechin, Trolox (6-hydroxy-2,5,7,8-tetramethylchroman-2-carboxylic acid), ABTS (2,2-azino-bis(3-ethylbenzothiazoline-6-sulfonic acid) diammonium salt), DPPH (2,2,-Diphenyl-1-picrylhydrazyl), aluminum chloride and sodium nitrite were supplied by Sigma-Aldrich (Milan, Italy). Sodium carbonate, sodium hydroxide, methanol, *n*-hexane, acetic acid, chloroform, diethyl ether, hydrochloric acid, phenolphthalein and potassium iodide was supplied by Carlo Erba (Milan, Italy).

### 2.2. Enrichment Matrix Analysis (Mace, Ginger and Turmeric)

Mace, ginger and turmeric were selected as enrichment matrices for their sensory attributes and high antioxidant potential. The characterization of raw materials was conducted as previously described [12,30,31].

### 2.3. Flavoured Olive Oil Analysis

Qualitative parameters were determined according to the EEC Regulation [32], such as free acidity (expressed as the % of oleic acid) and peroxide values (expressed as mEq O_2_/kg of oil).

For the extraction of the phenolics, the method previously described by Montedoro et al. [33] was applied. The oil was mixed with methanol (70%) and *n*-hexane. The resulting mixture was centrifuged, and the upper phase was collected, filtered and stored at −20 °C until analysis.

The total polyphenolic content (TPC) of EVOO and FOOs was determined following the methodology of Baiano et al. [34]. The total phenolic content was determined at 750 nm and expressed as mg GAE/kg of oil.

### 2.4. Breadstick Production Process

**Figure 1:**
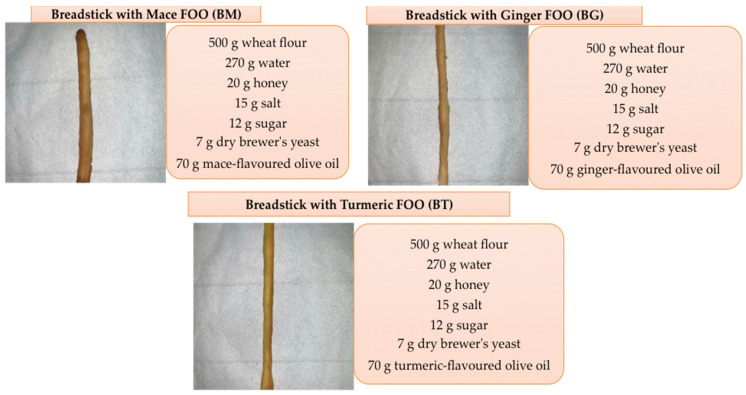
Formulation of enriched breadsticks.

The unflavoured olive oil used for the original recipe (EVOO) was extracted in an experimental olive oil mill at the University “Mediterranea” of Reggio Calabria, Italy, during the 2022 olive oil season. Flavoured olive oils (FOOs) were obtained by adding the matrices during the malaxation phase in a 1% ratio (10 g on 10 kg of olive paste). Breadsticks were prepared in a kitchen cooking processor (Kenwood, Havant, UK) with the following ingredients: 500 g wheat flour 00 type (55.93%), 270 g water (30.20%), 70 g oil (7.83%), 20 g honey (2.37%), 15 g salt (1.68%), 12 g sugar (1.34%) and 7 g dry brewer’s yeast (0.78%). Wheat flour, oil and salt were briefly mixed for 3 min at speed 1. The other ingredients were then added, with a slow constant addition of water at 30 °C while kneading the dough at speed 2. At the complete homogenization of the ingredients, the dough was then further kneaded for 10 min at speed 5. The dough was then left to rise for 9 h at 4 °C, and afterwards left at 18 °C for 2 h. Finally, the breadsticks were manually shaped, with dimensions of 1 × 0.5 × 10 cm (width × thickness × length), left to rest before cooking for 30 min and baked at 180 °C for 25 min (Figure 1), obtaining the following samples:

- The original recipe with the control EVOO (BCs);
- Breadsticks with a mace FOO (BMs);
- Breadsticks with a ginger FOO (BGs);
- Breadsticks with a turmeric FOO (BTs).

At the end of the cooking process, the breadsticks had a golden appearance and weighed between 15 and 18 g.

#### 2.4.1. Analysis of Breadsticks

##### Colour Analysis

The colour of breadsticks was determined with a reflectance colorimeter (Konica Minolta colorimeter, model CM-A177) following the CIE L* a* b* colour system. The lightness (L*) and colour parameters (+a*: red; −a*: green; +b*: yellow; −b*: blue) were assessed. The total colour difference (ΔE) between the control and enriched breadsticks was calculated using Equation (1):

ΔE = √(L2* − L1*) + (a2* − a1*) + (b2* − b1*)(1)

where L2*, a2* and b2* are the measured values of breadstick samples with flavoured olive oil, and L1*, a1* and b1* are the values of breadsticks made with the traditional recipe.

The colour was expressed by the chroma index (C*) [Equation (2)] and hue angle (H) [Equation (3)], which were used to evaluate the degree of saturation/fullness and the amount of redness and yellowness:

C* = √(a2 + b2)(2)

H* = arctan (b*/a*)(3)

##### Water Activity (a_w_)

Breadsticks were reduced to fine pieces through an electric grinder and sieved with an 800 μm sieve, and the water activity was measured with a LabMaster-a_w_ Novasina apparatus (Lachen, Switzerland) in three replicates.

##### Moisture Content (U.R.)

The moisture content was determined using a laboratory moisture analyser. An aliquot of the finely ground sample (5 g) was weighed and placed in the apparatus at 105 °C until the constant weight was reached. Each sample was analyzed in three replicates.

##### pH Determination

An aqueous solution made with 15 g of finely crushed breadsticks and 100 mL of H_2_O stirred for 30 min was prepared, filtered and fed through a pH meter in three replicates.

##### Total Phenol Content (TPC)

The extraction of the phenolic fraction was conducted following the method of Miskiewicz et al. [35], with modifications. Finely ground breadstick powder (5 g) was added to 2.5 mL of H_2_O, 20 mL of methanol and 0.25 mL of HCl; extraction was carried out for 30 min at 30 °C with a pulse mode of 2 s on/4 s off at 30% and with a frequency of 30 kHz. The solution obtained was then centrifuged at 6000 rpm for 10 min at 4 °C. The supernatant was collected, filtered through Whatman n. 4 filter paper and made up to a volume of 25 mL with MeOH/H_2_O (1:10, *v*/*v*).

The TPC was evaluated as previously described by Gonzalez-Molina et al. [36]. A 0.35 mL sample extract aliquot was mixed with 1 mL of Folin–Ciocalteu reagent, water and 10 mL of 20% Na_2_CO_3_ solution. Solutions were left for 2 h in the dark at room temperature and the absorbance was measured at 765 nm using a UV–Vis Agilent 8453 spectrophotometer (Agilent Technologies, Milan, Italy). The results were expressed as milligrammes of gallic acid equivalent per gram of fresh weight (mg GAE/g FW).

##### Total Flavonoid Content (TFC)

The TFC was determined following a flavonoid–aluminum complex methodology. The breadstick extract was mixed with 0.15 mL of 5% NaNO_2_ 5, 0.15 mL of 10% AlCl_3_ and 2 mL 1 M di-NaOH and H_2_O. The flavonoid content was determined using a (+)-catechin standard curve and expressed as the mean of the mg of (+)-catechin equivalents per gram of fresh weight (mg CE/g FW).

##### Antioxidant Capacity

A 1,1-Diphenyl-2-picryl-hydrazyl (DPPH) assay and a 2,2-azino-bis(3-ethylbenzothiazoline-6-sulfonic acid) (ABTS) assay were used to determine the free radical scavenging activity according to the previously published method [12].

In the DPPH radical scavenging test, a solution of DPPH was mixed with the extract (1–1000 µg/mL). The absorbance was read after 30 min of incubation at room temperature. In the ABTS test, a solution of an ABTS radical cation was prepared and diluted after 12 h with ethanol to reach an absorbance of 0.70 at 734 nm. The stabilized ABTS+ solution was then added to extracts (1–400 µg/mL) and the absorbance was read after incubation for 6 min at 25 °C.

##### Oxidative Stability

A rapid food stability measurement through oxygen uptake by subjecting the sample to a high temperature and pure oxygen in specific oxidation chambers was performed with an Oxitest apparatus (VELP Scientifica, Milano, Italy); a storage stability prediction for the shelf-life assessment was obtained using OxisoftTM software (Version 10002948 Usmate Velate, MB, Italy). A total of 30 g of breadstick samples was homogeneously placed into the instrument oxidation chambers and analyzed at an O_2_ pressure of 6 bar at 90 °C. The result of the Oxitest analysis was expressed as an induction period (IP), or the period before product fat oxidation. The analysis was conducted in duplicate.

##### Texture Analysis

The physical analysis of samples was carried out by means of a cut slice test and texture analysis using a TA-XT Plus Texture Analyzer (Stable Micro Systems Ltd., Godalming, UK). The data obtained were acquired and integrated using Exponent software, version 6.1.4.0 (Stable Micro Systems Ltd., UK).

Each sample was placed on a 3-point bend rig fixture (model A/3PB, Stable Micro Systems Ltd., UK) for hardness and factorability measurements. The product hardness (g) was measured at the maximum peak force; the product factorability (mm) was measured by the distance at breakup. The experimental conditions were as follows: the pre-test speed: 1 mm·s^−1^; the test speed: 3 mm·s^−1^; the post-test speed: 15 mm·s^−1^; and the distance: 10 mm. The analysis was conducted for two breakings of each of five breadsticks for each of three packages.

##### Sensory Evaluation

The sensory analysis was carried out through a quantitative descriptive analysis with a trained panel of 10 adults recruited among faculty staff, composed of 5 males and 5 females, aged between 23 and 65 years. Panel members assessed samples following ISO 8586:2012 [37] training for expert sensory assessors’ selection and training. The study did not reveal any personal information and/or images of participants and was conducted in accordance with the Declaration of Helsinki; panellists gave their informed consent for inclusion before they participated in the study.

The analysis was performed in sensory booths [38], with samples served in a random order at room temperature; between each sample, panellists cleansed their palate with natural mineral water. Tests were performed in triplicate and data were averaged. The results were expressed as the mean of a 10-point structured scale ranging from 0 (absence) to 9 (extremely high) for visual, olfactory, taste and texture descriptors.

#### 2.4.2. Analysis of Lipid Fraction

The extraction of the lipid fraction was conducted as suggested by Kozlowska et al. [39], with some modification. Finely ground breadstick powder (50 g) was weighed and homogenized with 250 mL of *n*-hexane and left under continuous stirring at ambient temperature (21 °C) for 40 min. The obtained mixtures were then left overnight at 4 °C to achieve phase separation and filtered. Finally, the solvent was evaporated through a rotary vacuum evaporator at 40 °C. The obtained fat fractions were frozen at −20 °C until analysis.

##### Physical–Chemical Characterization of Lipid Fraction Extracted from Breadsticks

The free acidity was determined according to the European Commission [32]. The lipid fraction was dissolved in a diethyl ether/ethanol solution (1:1, *v*/*v*) and the acidity was titrated with a 0.1 N NaOH solution with phenolphthalein as indicator. The acidity was expressed as the % of oleic acid by the formula proposed by the European Commission [29].

The determination of the peroxide value (PV) was carried out according to the European Commission [32]. A total of 1 g of the lipid fraction was weighed in a glass flask with a ground neck and stopper, and 25 mL of chloroform/acetic acid (3:2, *v*/*v*) was added. Next, 1 mL of a potassium iodide (KI) saturated solution was added, and the stopper was quickly inserted. After shaking for one minute, the sample was left for five minutes in the dark. At this point, 75 mL of deionised water and 6–7 drops of a 1% starch solution were added as an indicator. The determination was carried out by titrating the liberated iodine with the 0.01 N Na_2_S_2_O_3_ solution. The peroxide value (PV) was expressed in milliequivalents of active oxygen per kilogramme of oil (mEq O_2_/kg) by the formula proposed by the European Commission [32].

The *p*-anisidine value (NPA) was determined according to ISO standard methods (3960:2009, 6885:2008 and 3656:2011, respectively) [40,41,42]. A 2.5 g/L *p*-anisidine solution in glacial acetic acid was prepared. After that two solutions were prepared:

- Solution 1 (A1): weigh 0.5–4 g of the lipid fraction and make up to volume in a 25 mL flask with isooctane;
- Solution 2 (A2): take 5 mL of A1 and add 1 mL of the *p*-anisidine/acetic acid solution.

A1 and A2 were incubated for 10 min and read spectrophotometrically at 350 nm. The *p*-anisidine value was expressed using the following formula, Formula (4):

*p-AnV* = ((25 × (1.2 × (A_2_ − A_1_))/(sample weight)) (4)

##### Statistical Analysis

Results were expressed as the means of three different experiments ± the standard deviation (S.D.). All data were analyzed using a one-way analysis of variance (ANOVA) with SPSS 17.0 (SPSS Inc., Chicago, IL, USA) statistical software. Significant differences were calculated according to Tukey’s multiple range tests. Differences at * *p* < 0.05 were statistically significant. A principal component analysis (PCA) was also conducted using SPSS software for Windows, version 17.0 (SPSS, Chicago, IL, USA).

## 3. Results and Discussion

### 3.1. Colour Analysis

**Figure 2:**
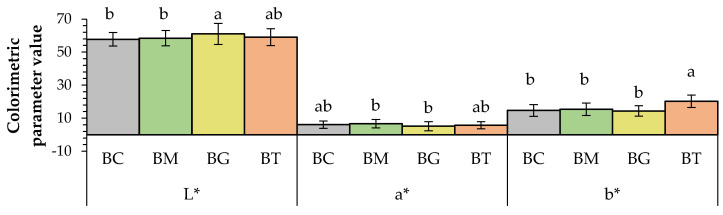
Colorimetric parameter values in breadsticks (L*, a*, b*). Data are expressed as means + S.D (*n* = 3). BC: control; BM: breadstick with mace-flavoured olive oil; BG: breadstick with ginger-flavoured olive oil; BT: breadstick with turmeric-flavoured olive oil. The lowercase letters indicate results significantly different (*p* < 0.01).

**Figure 3:**
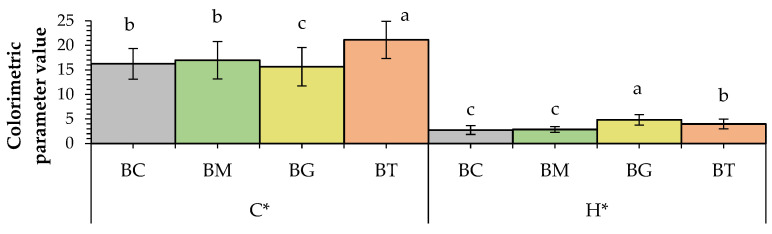
Chroma (C*) and hue angle (H*) of breadsticks. Data are expressed as means + S.D (*n* = 3). BC: control; BM: breadstick with mace-flavoured olive oil; BG: breadstick with ginger-flavoured olive oil; BT: breadstick with turmeric-flavoured olive oil. The lowercase letters indicate results significantly different (*p* < 0.01).

Colour is one of the primary parameters which affects consumers’ acceptability and influences the purchase of new products. Figure 2 and Figure 3 show the colour of breadsticks under evaluation. The L* value was the highest in breadsticks with ginger-flavoured olive oil (BGs) (61.02), proving to be the brightest, in contrast to the original recipe (BC), which was the darkest (57.78). The b* coordinate indicates shades from blue (−b*) to yellow (+b*). Examining the FOO colorimetric parameters [43], it is possible to evidence that turmeric FOO had significantly higher b* values than the other flavoured oils (2.20 vs. 2.12 and 2.14 for turmeric, mace and ginger FOOs, respectively). The same can be observed by analyzing the parameter C*, which considers the overall variation in colour (i.e., also a*) [44].

This shows how the “yellow” colour component derives mainly from the enrichment matrix. Turmeric is a spice known for its yellow colour, and this explains why in breadsticks (BTs) the b* value was the highest with an impact on cooking also determined in terms of the surface browning of the product. It is known that turmeric is characterized by marked yellowish shades. Before setting the cooking time, preliminary tests were conducted, until a uniform and golden surface was obtained. In the case of turmeric, its characteristic shades probably influenced the timing for achieving a uniformly coloured surface. In addition, other preliminary tests showed that if BTs were cooked for 25 min, like all the other formulations, they were not appreciated by the panellists. Moreover, other parameters such as the TPC, TFC or antioxidant activity were both tested at 21 and 25 min of cooking and the difference of only 4 min did not lead to significant differences in these parameters.

At the same time, the addition of mace-flavoured olive oil (BMs) resulted in an increase in the red shadows, showing the highest a* value of 6.66, followed by the BC sample with 6.12. Rainero et al. [45] fortified breadsticks with red grape pomace and noted how the a* value significantly increased, whereas b* decreased. By contrast, De Gennaro et al. [23] added olive cake powder to a breadstick formulation and the L* and b* values increased significantly as the concentration of the pomace powder increased. The overall effect on colour changes of the modified recipe was much more evident in the BT sample, given its C* value of 21.13, which is 30% higher than the BC’s (16.25). In the BG sample, it is worthwhile noting how the hue angle reached the highest value of 6.25, as much as 2.3 times higher than the BC’s (2.74), proving to be one of the most addition-influenced samples. The maximum total colour difference (ΔE) was obtained between BCs and BTs (2.54), while the lowest was found between BCs and BMs (1.39). The reason for this variation is probably ascribable to the raw materials used and the processing temperature [23,46].

### 3.2. Water Activity (a_w_), Moisture Content (U.R.) and pH

The water activity (a_w_) was mainly influenced by the storage temperature and packaging [47]. As shown in Table 1, there is a decrease (*p* < 0.05) in the water activity in the flavoured samples compared to the control, as well as in the pH values. Likewise, in the moisture content there are significant decreases (*p* < 0.01), except for BTs (7.92 vs. 8.12, for BCs and BTs, respectively) (Table 1). This could be due to the baking times; indeed, in the case of BTs it was slightly shorter (21 min). A positive Pearson’s correlation coefficient was found between the moisture content and the water activity a_w_ (*p* = 0.87). The values of the moisture found by Conte et al. [22] in the formulation of gluten-free breadsticks ranged from 7.66 to 11.50, significantly higher than ours (from 6.15 to 8.12). Hence, this formulation proved to have a greater quantity of water with consequent stability issues during storage, typical especially of gluten-free and starch-based products, in which it is more difficult for water to bind to starch due to the absence of gluten. Previously, Giuffrè et al. [29] replaced the use of extra virgin olive oil with olive pomace oil in the formulation of breadsticks. The results highlighted, similarly to our data, that the control breadstick had a higher moisture content than the new formulation. On the contrary, Petchoo et al. [48] highlighted a lower moisture content in functionalized breadsticks compared to the control, although with higher values than those obtained in this study (1.13–3.93 g/100 g).

### 3.3. TPC, TFC and Antioxidant Activity

Each flavouring matrix is characterized by considerable TPC values, especially mace (41.15 mg GAE/g FW), followed by turmeric with 29.65 and then ginger with 15.03 mg GAE/g FW (Table S1). Consequently, the total phenol content (TPC) in breadsticks significantly increased (*p* < 0.01) in the flavoured ones, compared to those prepared with unflavoured olive oil. This is due to the additive effect of the TPC of the individual matrices (spices), which led to an increase in the TPC in flavoured oils and consequently in flavoured breadsticks. The BM sample reached the highest content of 996.32 mg/kg, followed by the BT with 830.78 mg/kg, about 48.26 and 23.62% higher than the BC, respectively (Table 2). Similarly, Simsek et al. [24], replacing wheat flour with olive pomace powder at different concentrations, found TPC values higher than the control in a dose-dependent manner, with a range of values between 710 and 1070 mg GAE/kg [24]. Cedola et al. [49] enriched bread and spaghetti with olive paste and olive mill wastewater and revealed that all the new formulations showed higher levels than the control of about 12.9-fold in bread and 8.9 for spaghetti.

The total flavonoid content (TFC) is shown in Table 2; there was a strong variability among samples (*p* < 0.01). The control sample was characterized by a value of 249.26 mg CE/kg and, among the flavoured breadsticks, the most interesting value was represented by the BM sample (534.41 mg CE/kg), 2.15-fold higher than the BC, followed by the BG (448.12 mg CE/kg). In the BT sample, even lower TFC values than the BC were found (220.42 mg CE/kg), with a decrease of 11.57%. In data from raw enrichment matrices (Table S1), mace showed the greatest amount of these biomolecules with 26.98 mg CE/g FW vs. 16.01 in ginger and 17.41 in turmeric. Although in turmeric, the TFC was slightly higher than in ginger extract, in the breadsticks the flavonoid content was lower. The cooking process causes a significant decrease in bioactive compounds, as demonstrated by several authors. Indeed, Cedola et al. [50], while enriching “taralli” with olive leaf extract and analyzing products before and after the cooking process, observed a decrease in the TFC of about 8.33%. Similarly, Marinelli et al. [51], enriching pasta with grape pomace, despite having underlined how the enrichment increased the TFC thanks to the extrusion phase due to the release of tightly bound compounds, also highlighted how drying, pasteurization and cooking processes negatively influenced both the TPC and TFC. Probably, turmeric flavonoids are more prone to degradation at high cooking temperatures, which can justify why the BT samples presented even lower levels than the control. In fact, previously Chumroenphat et al. [52] showed that curcuminoids are particularly sensitive to the effect of temperatures and that they undergo degradation and transformation processes already at 50 °C.

The radical scavenging activity was studied through the DPPH and ABTS assays (Table 2). In the DPPH assay all the flavoured breadstick extracts showed a higher level than the control (268.94 vs. 393.91, 305.73 and 357.6 µM TEAC/100 g extract, for the BC, BM, BG and BT, respectively) an increment of 46.47, 13.67 and 32.97% for the BM, BG and BT from the BC, respectively. This potential was also confirmed by the data obtained from the ABTS assay in which, again, in all the flavoured breadstick extracts higher levels were evidenced than the control (94.06 vs. 125.04, 160.13 and 131.35 µM TEAC/100 g extract, for the BC, BM, BG and BT, respectively), an increment of 32.94, 70.24 and 39.65% for the BM, BG and BT from BC, respectively. The greater TPC and TFC in flavoured breadsticks is reflected in the increase in their radical scavenging potential since these compounds can exert their activity through two mechanisms, including hydrogen atom transfer or single electron transfer followed by proton transfer [53].

It is well known that phenols are positively correlated with antioxidant activities [54]; in fact, a high Pearson’s correlation coefficient was found at *p* = 0.96 between DPPH and the TPC. In this regard, in previous studies, the phenolic profiles of these flavoured oils were analyzed using UHPLC, highlighting positive correlations between the main compounds of turmeric (e.g., curcuminoids) and ginger (6-gingerol and 6-shogaol) that are strictly related to the antioxidant potential [30,31]. According to our data, Pasqualone et al. [18], who enriched biscuits with grape extract, observed an increase in radical scavenging activities.

### 3.4. Oxidative Stability

During the formulation of new baked foods, is very important to check the oxidative stability of the lipid fraction to predict the product’s shelf-life. The induction period (IP), which is an indication of the degree of oxidation, was obtained from the point of intersection of the straight line and the inflexion point of each test curve. Moreover, this point is consistent with the accelerated absorption of oxygen and the corresponding loss of protection against lipid oxidation. The results from the Oxitest analysis, observed in Figure S1, showed how the new formula’s breadsticks possessed a higher resistance to lipid oxidation than the control (IP of 53.19 h), with values of the IP of 92.44, 91.26 and 60.07 h, for the BM, BG and BT, respectively. Noteworthy is the highest IP registered in the BM and BG, with increases of 73.79 and 71.57% more than the BC, respectively. This greater oxidative stability could be attributable to the presence of antioxidants such as curcuminoids or other compounds with the same activities that can be isolated from ginger and mace. In fact, these compounds could inhibit lipid oxidation [11,12,13,55]. The Pearson’s correlation coefficient confirms that, as previously hypothesized, flavones from the flavouring matrix (turmeric, ginger and mace) play a key role in preserving the breadsticks from oxidation.

The free acidity significantly increased (*p* < 0.01) in mace- and ginger-flavoured breadsticks, with values 1.22- and 1.18-fold higher, respectively. On the contrary, in the BT sample the lowest acidity value was recorded, corresponding to 0.44% and −1.47-fold compared to the BC (Table 3). In contrast to these results, Giuffrè et al. [29] found lower free acidity values in breadsticks enriched with olive pomace oil compared to the formulation with EVOO. This is probably related to the rectification process applied to olive pomace oil before edible use. Furthermore, Giuffrè et al. [56], by modifying the original “cantuccini” and replacing cow’s butter and margarine with 100% EVOO, found a lower free acidity content of approximately 0.59%.

Lipid oxidation mainly occurs in high-moisture content or high-temperature conditions. The peroxide value in BC samples reached the highest values (8.28 mEq O_2_/kg), whereas all the flavoured breadsticks showed lower values, especially the BM (5.09 mEq O_2_/kg), about 1.62 times lower than the BC’s. This trend is totally opposite to that of unflavoured olive oil before cooking processes (Table S2). This means that the main molecules arising from each flavouring matrix play a crucial role in protecting the breadsticks from oil oxidation. Mildner-Szkudlarz et al. [57] formulated biscuits with green tea extract and demonstrated that the addition did not significantly influence the peroxide value (0.96 vs. 0.95 for the control and for the green tea biscuits, respectively). On the other hand, Giuffrè et al. [29] highlighted that in EVO oil breadsticks, unlike olive pomace oil breadsticks, the peroxide content was significantly lower by 1.38 times (4.41 vs. 6.10 mEq O_2_/kg, respectively). This confirms that the high presence of bioactive compounds counteracts the oxidation of the oil.

In relation to the *p*-anisidine value (NPA), which measures the secondary oxidation rate, a general increasing trend was observed in the new breadsticks. The use of FOOs did not appear to improve the secondary lipid stability, especially in the BG sample, where the highest value of 21.08 was recorded, approximately +94.64% higher than the BC’s.

The totox (OX) describes the degree of oxidation of fat and is generally used to measure the non-volatile carbonyl precursors of the lipid fraction and the development of further oxidation compounds [57].

The totox of BC samples was significantly (*p* < 0.01) lower than that of the BM, BG and BT with values of 13.12 vs. 18.16, 27.18 and 22.56, respectively. Mildner-Szkudlarz et al. [57] noted higher totox values only in the combination of biscuits with 1% of a green tea leaf extract. After a 20-day storage period, the values were about 61% lower than the control (29.73 vs. 11.40, respectively). The individual bioactive molecules exerted different actions in the formation of primary and secondary oxidation products, such as alpha-tocopherols and catechins [57]. Hence, researchers hypothesized that antioxidant activity is influenced by a complex phenomenon in which the polarity of each bioactive compound is not the only parameter to be considered.

### 3.5. Texture Analysis

The texture behaviour of modified products showed a significant difference (*p* < 0.01) for the hardness parameter, one of the key parameters for products like breadsticks; the hardness was higher in the case of the ginger breadstick (BG) and lower in the case of the mace breadstick (BM). In the case of the BG, this increase in the experimental force required at break can be explained by its lower U.R. and a_w_ [22].

Minor but still significant differences (*p* < 0.05) were found for the fracturability parameter, where ginger and turmeric breadsticks showed slightly higher values compared to the control (Table 4). However, due to the formulation of the new product, the assessment of the correlation between this parameter and the moisture content can be difficult, as in other baked snacks [22].

### 3.6. Sensory Analysis

**Figure 4:**
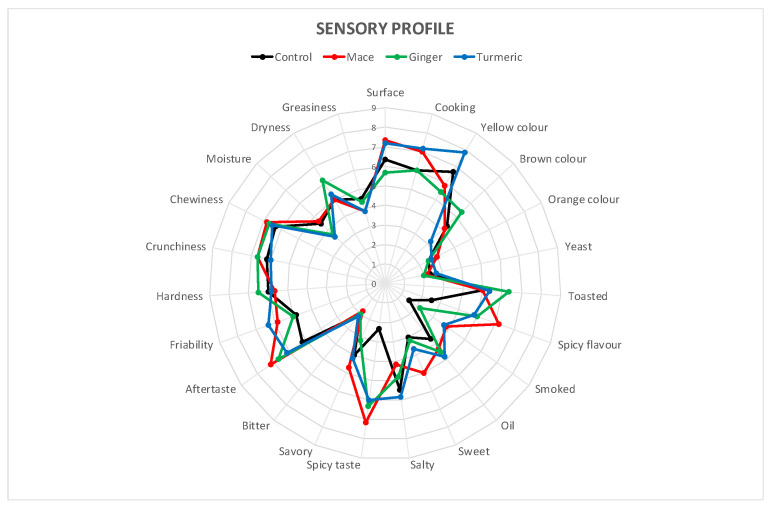
Sensory profile of breadsticks.

The main appearance descriptors were found to be the type of surface and the cooking intensity, along with the presence of a yellow colouring; the main olfactory descriptors were a toasted, spicy and oily flavour, while the principal taste descriptors were a spicy and salty taste, along with the general aftertaste intensity; the textural descriptors were all actively recognized by panellists, except for the moisture and greasiness descriptors. Mace breadsticks differentiated significantly from the control with higher values in almost all descriptors, while turmeric breadsticks showed higher descriptor values in the appearance and texture descriptors. On the other hand, ginger breadsticks did not differentiate significantly from the control, apart from in a few descriptors, like a brown colour, toasted flavour and a spicy flavour and taste (Figure 4).

### 3.7. Principal Component Analysis (PCA)

In this study, a Pearson correlation test was performed to evaluate the relationship between the different parameters evaluated (Table S4). A high correlation was observed between the DPPH assay and the total phenol content and for the ABTS assay with the oxidative stability (OX). Other correlations were observed between b* and C* and between L* and H*, OX and ABTS. The peroxide value showed a good correlation with L*, C*, the TPC, TFC, OX, ABTS and DPPH. The acidity value showed a high correlation with the total flavonoid content (TFC). No correlation was found between the oxidative stability (OX) and the TPC and TFC, as would be expected.

Principal component analysis (PCA) is the statistical tool used to explain differentiation between samples and to obtain more information on the main variables that influence the samples’ similarities and differences.

**Figure 5:**
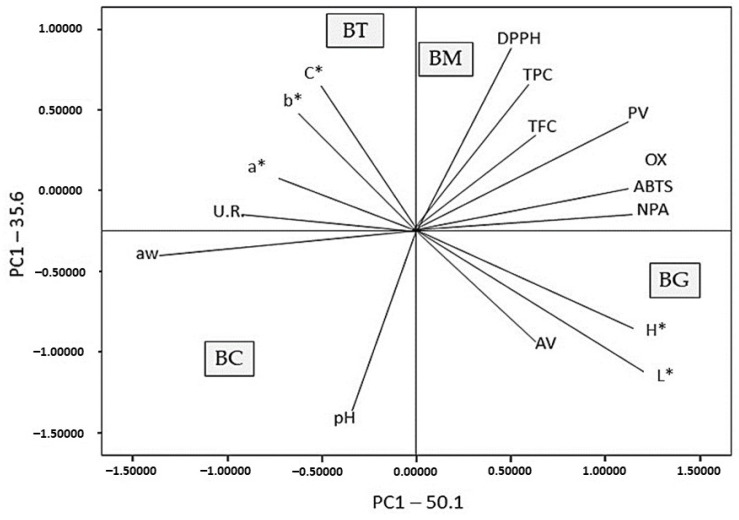
Biplot of the two most significant principal components, PC1 vs. PC2, identified by the principal component analysis (PCA) of the analytical parameters of the control sample and the flavoured samples.

Figure 5 demonstrates the interrelation among the analyzed parameters and the positioning of the analyzed flavoured breadsticks in comparison to each other.

A PCA was performed on the whole set of average values with a variance of 50.1% in the first principal component (PC1) and 35.6% in the second (PC2) (Figure 5). The PCA showed that the two principal components accounted for 85.7% of the total variance. Thus, these components can be used to represent the set of variables measured in the packages tested.

The first principal component (Figure 5) shows a strong positive correlation with the PV, NPA, L*, TFC, OX and ABTS. The significant correlations obtained support the hypothesis that the TFC contributes significantly to the antioxidant activity, especially for the ABTS assay and the oxidative stability (OX). In addition, from the analysis of variable loads, it is seen that the PC1 has a negative correlation with the pH, aw, U.R., a*, b* and C*. The second principal component is well correlated with b*, C*, the TPC and DPPH. In the PC2, the TPC contributes significantly to the antioxidant activity, especially for the ABTS assay.

Polyphenolic compounds (the TPC and TFC) are grouped on the right side of the PCA plot; these factors indicate that a higher antioxidant biomolecule content in samples determines greater antioxidant activity and greater oxidation stability.

The BM is positively correlated with the TPC, TFC and DPPH, while the BG has a higher antioxidant activity in reference to the ABTS assay and oxidative stability compared to the BM and BT samples. From the PCA graph, it can be observed how the BT, BM and BC samples show higher bioactivity compared to the BC control sample, in addition to having a higher oxidative stability.

## 4. Conclusions

This study explored the effect of the addition of flavoured olive oils (turmeric, mace and ginger) on the chemical composition and textural, sensory and biological properties of breadsticks, a typical Italian product. The substitution of the EVOO with flavoured olive oil influenced the colorimetric parameters C* and L*, especially for the breadsticks enriched with ginger and mace (BGs and BMs). Because of the increase in the free acidity of the FOO, an increase in peroxide values was observed in all enriched breadsticks. Furthermore, all flavoured breadsticks proved more resistant to lipid oxidation, with a positive impact on their shelf-life. The content of phenolic and flavonoid compounds was also interesting, as well as the radical scavenging activities, with small differences between the samples examined. In fact, the replacement of flavoured oils in the traditional recipe increased the content of bioactive compounds in the breadsticks, as demonstrated by the increase in the TPC and TFC, as well as by the increase in the free radical scavenger activity, exerted especially by the BM and BG.

Texture analysis revealed a different structural behaviour between samples, with significantly higher hardness values for the ginger breadstick and lower values for the mace-flavoured breadstick. Ginger and turmeric samples, on the other hand, showed different behaviour regarding the fracturability parameter, thus requiring a sensory textural assessment for the identification of the sample with the most improved textural characteristics.

The sensory analysis identified the BG as the breadstick with the greatest toasting intensity while the BM showed spiciness characteristics. The BC was also found to be the crumbliest breadstick, while greater crunchiness was found in the BG and BM samples. The overall sensory results showed values without high differences between the control and flavoured products; this result, in association with the low scores found in negative descriptors such as a bitter taste and texture, moisture and greasiness, contributed to not influencing the product acceptability for the panel members. Therefore, all products were rated positively, with the best result achieved by replacing EVOO with mace-flavoured olive oil (BM).

## Figures and Tables

**Table 1:** Main qualitative parameters of breadsticks.

Sample	pH	a_w_	Moisture (U.R.) (%)
BC	5.86 ± 0.12 ^a^	0.58 ± 0.02 ^a^	7.92 ± 0.35 ^b^
BM	5.74 ± 0.11 ^b^	0.50 ± 0.01 ^ab^	6.18 ± 0.42 ^c^
BG	5.80 ± 0.21 ^ab^	0.45 ± 0.01 ^b^	6.15 ± 0.27 ^c^
BT	5.78 ± 0.34 ^ab^	0.54 ± 0.02 ^a^	8.12 ± 0.26 ^a^
Sign	*	*	**

Data are expressed as means + S.D (*n* = 3). BC: control; BM: breadstick with mace-flavoured olive oil; BG: breadstick with ginger-flavoured olive oil; BT: breadstick with turmeric-flavoured olive oil. Results followed by different letters in each column are significantly different (** *p* < 0.01; * *p* < 0.05).

**Table 2:** Bioactive compound content (TPC and TFC) and radical scavenging potential of breadsticks.

	TPC (mg/kg)	TFC (mg/kg)	ABTS (µM TEAC/100 g Extract)	DPPH (µM TEAC/100 g Extract)
BC	672.01 ± 4.78 ^d^	249.26 ± 3.89 ^c^	94.06 ± 9.67 ^d^	268.94 ± 18.52 ^d^
BM	996.32 ± 3.93 ^a^	534.41 ± 6.84 ^a^	125.04 ± 12.78 ^c^	393.91 ± 21.54 ^a^
BG	783.48 ± 4.67 ^c^	448.12 ± 4.85 ^b^	160.13 ± 9.89 ^a^	305.73 ± 19.74 ^c^
BT	830.78 ± 4.35 ^b^	220.42 ± 5.48 ^d^	131.35 ± 14.87 ^b^	357.6 ± 12.58 ^b^
Sign	**	**	**	**

Data are expressed as means + S.D (*n* = 3). BC: control; BM: breadstick with mace-flavoured olive oil; BG: breadstick with ginger-flavoured olive oil; BT: breadstick with turmeric-flavoured olive oil. Results followed by different letters in each column are significantly different (** *p* < 0.01).

**Table 3:** Main qualitative parameters of breadsticks.

	Free Acidity (% Oleic Acid)	Peroxide Value (mEq O_2_/kg)	*p*-Anisidine Value	Totox
BC	0.65 ± 0.03 ^b^	8.28 ± 0.08 ^a^	10.83 ± 0.56 ^d^	13.12 ± 0.23 ^d^
BM	0.77 ± 0.02 ^a^	5.09 ± 0.12 ^c^	13.06 ± 0.63 ^c^	18.16 ± 0.89 ^c^
BG	0.79 ± 0.04 ^a^	6.09 ± 0.22 ^b^	21.08 ± 0.28 ^a^	27.18 ± 0.69 ^a^
BT	0.44 ± 0.01 ^c^	5.71 ± 0.18 ^c^	16.84 ± 0.76 ^b^	22.56 ± 0.76 ^b^
Sign	**	**	**	**

Data are expressed as means + S.D (*n* = 3). BC: control; BM: breadstick with mace-flavoured olive oil; BG: breadstick with ginger-flavoured olive oil; BT: breadstick with turmeric-flavoured olive oil. Results followed by different letters in each column are significantly different (** *p* < 0.01).

**Table 4:** Data on texture analysis.

	BC	BM	BG	BT	Sign
Hardness	1503.3 ± 35.71 ^bc^	1073.53 ± 28.60 ^c^	2174.5 ± 30.42 ^a^	1568.61 ± 42.49 ^b^	**
Fracturabiliy	−80.31 ± 0.76 ^b^	−80.49 ± 0.75 ^b^	−79.85 ± 0.54 ^a^	−78.99 ± 1.63 ^a^	*

Data are expressed as means + S.D (*n* = 3). BC: control; BM: breadstick with mace-flavoured olive oil; BG: breadstick with ginger-flavoured olive oil; BT: breadstick with turmeric-flavoured olive oil. Results followed by different letters in each row are significantly different (** *p* < 0.01; * *p* < 0.05).

## Data Availability

The original contributions presented in this study are included in the article and its Supplementary Materials. No data were deposited in any publicly available repositories. Further inquiries can be directed to the corresponding authors.

## Supplementary Materials

The following supporting information can be downloaded at https://www.mdpi.com/article/10.3390/antiox13121438/s1: Table S1: Total phenol content (TPC) and total flavonoid content (TFC) of enrichment matrices; Table S2: Main qualitative parameters of EVOO and FOOs used for breadstick preparation; Table S3: Sensory analysis of breadstick samples; Table S4: Correlation matrix. Figure S1: Induction period of breadstick samples.
