# Supplementary material for: Enrichment of Breadsticks with Flavoured Oils: Chemical Composition, Antioxidant Activity and Technological and Sensory Properties
Source: Antioxidants (Basel). 2024 Nov 22;13(12):1438. doi: 10.3390/antiox13121438 (PMC11672860; doi:10.3390/antiox13121438)
Supplement: Supplementary file 1 [file antioxidants-13-01438-s001.zip › antioxidants-3277132-supplementary.pdf]

Table S1. Total phenol content (TPC) and total flavonoid content (TFC) of enrichment matrices.

	TPC (mg/g)	TFC (mg/g)
**Mace**	41.15 ± 4.77[a]	26.98 ± 1.33[a]
**Ginger**	15.03 ± 2.03[c]	16.01 ± 2.4[b]
**Turmeric**	29.65 ± 3.41[b]	17.41 ± 3.21[b]
**Sign.**	**	*

Data are expressed as mean + S.D. ($n = 3$). Results followed by different letters in each column are significantly different (**$p < 0.01$; *$p < 0.05$).

Table S2. Main qualitative parameters of EVOO and FOOs used for breadsticks preparation.

	Free acidity (% oleic acid)	Peroxide Value (mEq O_2/kg)	TPC
**EVOO**	0.41 ± 0.03[d]	10.56 ± 0.21[b]	796.34 ± 18.44[b]
**M-FOO**	0.93 ± 0.01[b]	11.18 ± 0.50[a]	952.79 ± 21.71[a]
**G-FOO**	0.97 ± 0.02[a]	9.5 ± 1.02[c]	706.21 ± 1.47[bc]
**T-FOO**	0.52 ± 0.02[c]	10.39 ± 0.52[b]	953.39 ± 5.61[a]
**Sign**	**	**	*

Data are expressed as mean + S.D. ($n = 3$). EVOO: control; M-FOO: Mace flavoured olive oil; G-FOO: Ginger flavoured olive oil; T-FOO: Turmeric flavoured olive oil. Results followed by different letters in each column are significantly different (**$p < 0.01$; *$p < 0.05$).

Table S3. Sensory analysis of breadstick samples.

	BC	BM	BG	BT
Appearance descriptors				
**Surface**	6.3	7.3	5.7	7.2
**Cooking**	6.0	7.0	6.0	7.2
**Yellow color**	6.7	5.8	5.5	7.8
**Brown color**	4.3	4.2	5.3	3.2
**Orange color**	2.5	3.0	2.5	2.7
Olfactory descriptors				
**Yeast**	2.3	2.2	2.0	2.7
**Toasted**	5.0	5.0	6.3	5.3
**Spicy flavor**	2.5	6.2	5.0	4.8
**Smoked**	1.5	3.8	2.2	3.7
**Oil**	3.7	4.3	4.5	4.8
Taste descriptors				
**Sweet**	3.0	5.0	3.2	3.7
**Salty**	5.5	4.2	4.8	5.8
**Spicy taste**	2.3	7.2	6.3	6.0

**Savory**	4.0	4.7	3.2	4.2
**Bitter**	2.0	1.8	2.0	2.2
**Aftertaste**	5.2	7.2	6.7	6.2
Textural descriptors				
**Friability**	4.8	5.8	5.0	6.3
**Hardness**	6.0	5.7	6.5	5.8
**Crunchiness**	6.2	6.7	6.7	6.0
**Chewiness**	6.3	6.8	6.7	6.5
**Moisture**	4.5	4.7	3.7	3.5
**Dryness**	5.0	5.0	6.2	5.3
**Greasiness**	4.5	3.8	4.3	3.8

BC: control; BM: breadsticks with mace flavoured olive oil; BG: breadsticks with ginger flavoured olive oil; BT: breadsticks with turmeric flavoured olive oil.

Table S4. Correlation matrix

	pH	aw	U.R.	A.V.	P.V.	L*	a*	b*	C	H	TPC	TFC	OX	ABTS	DPPH
pH	1														
aw	0.527	1													
U.R.	0.492	0.867	1												
A.V.	-0.074	-0.61	-0.883	1											
P.V.	-0.741	-0.798	-0.473	0.03	1										
L*	0.058	-0.804	-0.584	0.522	0.512	1									
a*	-0.272	0.429	0.006	0.075	-0.445	-0.801	1								
b*	-0.35	0.268	0.591	-0.9	0.367	-0.404	-0.077	1							
C	-0.356	0.311	0.606	-0.904	0.324	-0.466	-0.008	0.998	1						
H	-0.046	-0.766	-0.406	0.232	0.681	0.942	-0.909	-0.074	-0.143	1					
TPC	-0.963	-0.443	-0.548	0.212	0.555	-0.177	0.491	0.177	0.199	-0.148	1				
TFC	-0.59	-0.758	-0.97	0.848	0.401	0.371	0.228	-0.545	-0.544	0.188	0.688	1			
OX	-0.407	-0.816	-0.42	0.081	0.912	0.776	-0.763	0.21	0.148	0.917	0.189	0.259	1		
ABTS	-0.487	-0.917	-0.598	0.265	0.921	0.807	-0.664	0.064	0.006	0.894	0.307	0.452	0.978	1	
DPPH	-0.969	-0.305	-0.324	-0.06	0.582	-0.303	0.456	0.436	0.457	-0.188	0.962	0.47	0.198	0.267	1

.

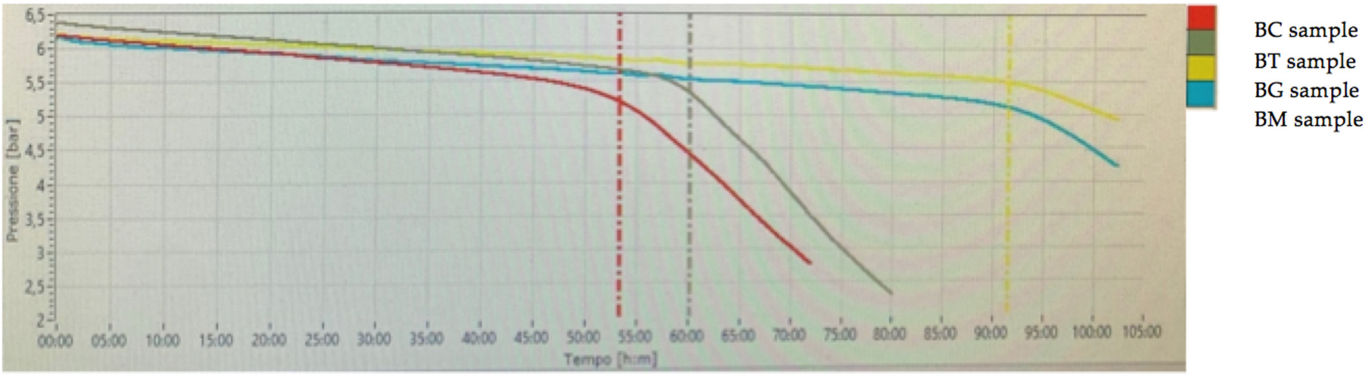

Figure S1. Induction period of breadstick samples
